# Outcome of tailored antiplatelet therapy in carotid stenting: a retrospective comparative study

**DOI:** 10.1186/s42155-024-00482-2

**Published:** 2024-10-09

**Authors:** Pavol Vigláš, Vojtěch Smolka, Jan Raupach, Aleš Hejčl, David Černík, Filip Cihlář

**Affiliations:** 1 Deptartment of Radiology, Faculty of Medicine in Hradec Králové, Charles University, Hradec Králové, Czech Republic; 2 Department of Radiology, University Hospital Hradec Králové, Hradec Kralove, Czech Republic; 3 Deptartment of Radiology, Masaryk hospital - J.E. Purkinje University and KZ a .s., Ústí nad Labem, Czech Republic; 4 Department of Neurosurgery Masaryk Hospital -J.E. Purkinje University and KZ a.s., Ústí nad Labem, Czech Republic; 5 Deptartment of Neurology, Masaryk Hospital - Ústí nad Labem, Czech Republic

**Keywords:** Carotid stenosis, Carotid stenting, Antiplatelet therapy

## Abstract

**Background:**

Carotid stenting requires dual antiplatelet therapy to effectively prevent thromboembolic complications. However, resistance to clopidogrel, a key component of this therapy, may lead to persistent risk of these complications. The aim of this study was to determine, if the implementation of routine platelet function testing and adjusting therapy was associated with lower incidence of thromboembolic complications and death.

**Methods:**

All consecutive patients treated with carotid artery stenting in a single institution over 8 years were enlisted in a retrospective study. Platelet function testing was performed, and efficient antiplatelet therapy was set before the procedure. Incidence of procedure-related stroke or death within periprocedural period (0–30 days) was assessed. The results were evaluated in relation to the findings of six prominent randomized control trials.

**Results:**

A total of 241 patients were treated for carotid stenosis, seven patients undergo CAS on both sides over time. There was 138 symptomatic (55,6%) and 110 asymptomatic stenoses (44,4%). Five thromboembolic complications (2,01%) occurred, four of them (1,61%) was procedure-related. Two patients died because of procedure-related stroke (0,82%). Incidence of procedure-related stroke or death was significant lower compared to the results of CREST study (2,01% vs. 4,81%, *P* = 0,0243) in the entire cohorts, and to the results of ICSS study in the symptomatic cohorts (2,86% vs. 7,37%, *P* = 0,0243), respectively.

**Conclusions:**

Tailored antiplatelet therapy in carotid stenting is safe and seems to be related with lower incidence of procedure-related death or stroke rate. Larger prospective studies to assess whether platelet function testing-guided antiplatelet therapy is superior to standard dual antiplatelet should be considered.

## Introduction

Stroke is the second leading cause of death and the leading cause of disability worldwide. Atherosclerotic stenosis of the internal carotid artery (ICA) is responsible for approximately 20% of ischemic strokes [1, 2]. The first carotid endarterectomy was performed by DeBakey in 1953. Endovascular methods for the treatment of carotid stenosis have been available since the early 1980s. Firstly in the form of angioplasty for non-atherosclerotic ICA lesions. Treatment of atherosclerotic ICA stenosis has been used since the early 1990s [3].

Carotid artery stenting (CAS) is currently a proven method of treatment for carotid stenosis. CAS is a safe alternative to surgical endarterectomy with comparable long-term results [2, 4–9]. The introduction of new technologies and the better selection of appropriate patients helps to reduce the incidence of periprocedural complications. However, periprocedural risks associated with CAS persist [4, 10–12]. Among the most serious complications of CAS is stroke. Most strokes are ischemic, hemorrhagic strokes are less commonly reported [13]. Other complications include transient ischemic attack (TIA), myocardial infarction (IM), hypotension, bradycardia, and bleeding from the vascular access [6].

Dual antiplatelet therapy (DAPT) significantly reduces the incidence of periprocedural thromboembolic complications [14–19]. DAPT consists of acetyl-salicylic acid (ASA) a cyclooxygenase-1 inhibitor and a second drug referred as a P2Y_12_ receptor inhibitor. The most common combination is ASA and clopidogrel. Multiple studies in recent years have reported the incidence of clopidogrel resistance in cohorts of patients undergoing neuroendovascular procedures, as well as a higher incidence of thromboembolic complications in these patients [14–19]. The incidence of clopidogrel resistance varies from 21 to 55% according to the literature [20, 21]. Several studies show ticagrelor as a drug with lower resistance rates (up to 10%) that better inhibits platelet function in vitro, including clopidogrel-resistant patients [15, 16, 20]. They also describe the use of ticagrelor as a safe alternative with similar efficacy in preventing thromboembolic events [15, 16, 18]. The incidence of ASA resistance ranges from 5 to 56% [20, 22]. The interindividual variability in response to antiplatelet drugs is most often due to genetic receptor polymorphisms, interactions with other drugs, and comorbidities. Significant reduction of clopidogrel effect is caused by e.g. proton pump inhibitors, in the case of ticagrelor by concomitantly administered corticosteroids. Finally, patient compliance plays an important role [21–23].

## Methods

In this study, we primarily analyze outcome of patients who undergo carotid stenting from 1st January 2014 to 31^st^ December 2022 at the Radiology department of Masaryk Hospital in Ústí nad Labem, Czech Republic (MNUL). Secondarily, results from MNUL were compared to results of prominent randomized controlled trials (RCT), namely CREST [Stenting versus Endarterectomy for Treatment of Carotid-Artery Stenosis], ICSS [Carotid artery stenting compared with endarterectomy in patients with symptomatic carotid stenosis (International Carotid Stenting Study)], ACST—2 [Second asymptomatic carotid surgery trial: A Randomized Comparison of Carotid Artery Stenting versus Carotid Endarterectomy], ACT—1 [Randomized Trial of Stent versus Surgery for Asymptomatic Carotid Stenosis], SPACE-2 [Angioplasty in asymptomatic carotid artery stenosis vs. endarterectomy compared to best medical treatment] and SAPPHIRE [Protected Carotid-Artery Stenting versus Endarterectomy in High-Risk Patients] [5–9, 24]

### Study criteria

The majority of stenoses were diagnosed by Doppler ultrasound and then confirmed by CT angiography. The degree of stenosis was determined according to NASCET (North American Symptomatic Carotid Endarterectomy Trial) criteria [4]. Carotid intervention was considered in symptomatic patients if the degree of stenosis was between ≥ 50% and ≤ 99%, and in asymptomatic patients between ≥ 70% and ≤ 99%. Assessment of clinical symptomatology was performed by the referring neurologist or neurosurgeon. Regular follow-up of patients after CAS was conducted by neurologist or neurosurgeon in outpatient clinic and is based on control ultrasound. We considered symptomatic patients as those with evidence of cerebral ischemia or amaurosis fugax in the previous six months. Patients with high surgical risk for surgery or surgically intractable stenosis were referred for primary CAS after consultation with a neurosurgeon and radiologist. Contraindications to stenting in the indication analysis were marked tortuosity of the arteries, inability to achieve safe vascular access, and a circularly calcified lesion. Four patients, who did not successfully undergo stent implantation due to technical complications, were excluded from the study. Typically, when the common carotid artery was excessively coiled, the proximal ICA was distressed to the point where the procedure had to be stopped after the guiding catheter was inserted. No complications were noted in these four patients. Patients treated with CAS for other reasons (tandem occlusion, carotid dissection) were not included in this study.

### Antiplatelet therapy regimens

DAPT was initiated at least 5 days prior to efficacy testing at doses of 100 mg ASA and 75 mg clopidogrel daily. After this period, all patients were tested for efficacy of antiplatelet therapy as part of the preoperative examination or at admission. In vitro platelet function was determined by certified laboratory methods using light transmission aggregometry (*n* = 169) or the Multiplate® analyzer (*n* = 79), as appropriate. When clopidogrel resistance was detected, treatment was changed to ticlopidine, which was taken at least five days before the procedure at a dose of 2 × 250 mg daily. Laboratory effectiveness of ticlopidine was tested after this period. After its withdrawal from the market in 2020, it was replaced by ticagrelor. In the case of ticagrelor, a loading dose of 2 × 90 mg was given, and the platelet function test was repeated the next day. In the case of resistance to both ticlopidine and ticagrelor, the loading dose 2 × 30 mg of prasugrel was given, and procedure was performed the next day. Platelet function test was not performed during follow-ups after CAS. The use of DAPT was recommended for a minimum of 1 month after the procedure at daily maintenance doses of 100 mg ASA in combination with 75 mg clopidogrel, or 90 mg ticagrelor twice daily, or prasugrel 5 mg daily. ASA monotherapy was continued after the completion of DAPT. The efficacy of ASA for completing the overall picture of platelet function was also routinely tested. ASA is currently used as a permanent component of DAPT. So far, higher doses of ASA have not been shown to reduce the incidence of thromboembolic events. Also, there is no alternative drug available to replace the effects of ASA [22]. Therefore, our focus was only on clopidogrel alternatives.

### Platelet function tests details

Light transmission aggregometry (LTA) is the oldest and well-established method for assessing platelet function [19, 20, 22]. LTA measurements were performed in platelet-rich plasma (PRP) using the turbidimetric method on an APACT 4004 aggregometer (LABiTec, Ahrensburg, Germany). The non-clotting blood sample was collected into 0.109 M sodium citrate in a 1:9 ratio (1 part citrate + 9 parts blood). Subsequently, the sample was centrifuged for 10 min at 150 g. To prepare platelet-poor plasma (PPP) for the blank, the sample was centrifuged for 10 min at 2 500 g. The platelet count was determined from the obtained PRP, and the optimal range for platelet count in PRP was determined to be 150–600 × 10^9^/L. If the platelet count was > 600 × 10^9^/L, platelet count was adjusted to 350 × 10^9^/L using saline. If the platelet count was < 150 × 10^9^/L, the result was issued with a comment: platelet aggregation may be falsely influenced by low platelet count. The inducers used for LTA measurements were adenosine diphosphate (ADP) at a concentration in the cuvette of 4 umol/L and arachidonic acid (ACA) at a concentration in the cuvette of 1 mmol/L (Hyphen BioMed, Paris,France). For the actual measurement, 140 ul of citrate PRP was pipetted into a tempered measuring cuvette at 37 °C with a magnetic stirrer inserted. Aggregation was initiated by adding 20 ul of ADP or ACA with constant stirring at 250 g. During the formation of plate aggregates, the PRP gradually cleared in the cuvette and light transmission rose, the change of which was recorded in the aggregation curve. Aggregation was measured for 10 min. The aggregation results were given as the maximum amplitude in %. The efficacy of antiplatelet therapy for patients treated with acetylsalicylic acid using an arachidonic acid (ACA) inducer of 1 mmol/L was determined to be in the range of 0–20%. Treatment with clopidogrel or ticagrelor was judged to be effective at Amax below the reference range for untreated patients, which is 60–104% for ADP /with a dose of the ADP agonist used of 4 umol/L. [20].

Multiplate® analyzer (Roche Diagnostics, Mannheim, Germany) detects platelet aggregation by measuring impedance changes. Analysis begun with filling 1.6 ml blood into a hirudin coated tube (Monovette-S, Sarstedt, Nümbrecht, Germany) and rested at room temperature. Platelet inhibition was measured by ASPI and ADP test using the Multiplate Analyzer according to the manufacturer’s instructions. 300 ul of hirudin blood was first diluted with prewarmed (37 °C) isotonic sodium chloride. After an incubation period of 3 min at 37 °C, 20 ul of the corresponding agonist (arachidonic acid (AA) or adenosine diphosphate (ADP) was added to each. Only commercially available standard reagents were used (Roche Diagnostics, Mannheim, Germany). The actual measurement time for each test was 6 min. The detected aggregation was expressed as aggregation units (AU), aggregation velocity (AU/min) and area under the curve [AUC, AU*min (U)]. For further analysis, the AUC was chosen as output parameter. With utilizing a hirudin coated tube, thresholds were < 45 U for ADP and < 30 U for ASPI test. In our region LTA and Multiplate® are available in many general hospitals and in all cerebrovascular centers where CAS is performed.

### Carotid stenting procedure

The actual CAS procedure was performed on a Philips Allura Xper FD 20 monoplanar angiography machine [Philips, Best, The Netherlands]. After 11 cm long 5F Avanti + sheath inducer [Cordis, USA] was inserted in the femoral artery, the common carotid artery was selectively catheterized using angled diagnostic catheter over 0.035" wire and left anterior oblique projection on the fluoroscopic system. Diagnostic catheter was selected based on anatomical properties, the most used was VER 135° [Cordis, USA]. Selective angiography of the common carotid artery in multiple projections was performed to verify carotid artery stenosis. Posteroanterior and lateral DSA projection including entire skull was mandatory, arteriography with oblique projection was performed when better carotid bifurcation visualization was needed. The injection rate of contrast medium using contrast pump injector was 4 ml/s. The guide wire was then advanced into the external carotid artery followed by advancement of the catheter into the external carotid artery. After the diagnostic part of the procedure, 5F sheath was exchanged for a 11 cm long 6F Avanti + sheath inducer [Cordis, USA]. Over a stiffer 0.035 wire" a guiding sheath was advanced into the common carotid artery.

The most used guiding sheath was 90 cm long Destination [Terumo, USA] (*n* = 191) with inner diameter (ID) 2.1 mm and outer diameter (OD) 2,8 mm, and 90 cm long Shuttle [COOK, USA] (*n* = 35) with ID 2,2 mm and OD 2,6 mm. Envoy with length of 100 cm [Cerenovus, USA] (*n* = 16) ID 1,8 mm and OD 2 mm and 80 cm long Asahi Fubuki [Asahi Intecc., JAP] (*n* = 6) with ID 1,8 mm, OD 2 mm were less frequently used. During each procedure we administered heparin at the dose of 70 units/kg IV bolus, the maximum dose was 5000 units. We didn’t use protamine after the procedure.

Filter Wire EZ [Boston Scientific, USA] on a 0.014" wire was used as a distal thromboembolic protective device. Filter wire was inserted by traversing a wire through the lesion and placing the filter distally using a delivery sheath, where withdrawal of the sheath deploys the filter. The filter catches emboli from atherosclerotic plaques larger than the 110-micron pore size. Filter retrieval was performed via separate retrieval sheath. The actual stenosis treatment was performed according to the operator's choice. When diameter of ICA in the stenosis was 1 mm or less, predilatation was performed to ensure adequate stent expansion needed for safely extraction of delivery system. Savvy [Cordis, USA] and Trek [Abbott, USA] balloons with the diameter of 2 or 3 mm were used for predilatation in 181 patients. A self-expandable carotid stent was then inserted; the dominant stents used were Wallstent [Boston Scientific, USA] in 112 patients, Precise [Cordis Corporation, USA] in 77 patients, and Adapt [Boston Scientific, USA] in 17 patients. The double-layered CGuard stent [InspireMD Inc., Tel Aviv, Israel] was used in 15 patients. The other stents were Acculink [Abbott Vascular, USA], Cristallo [Invatec, Italy], Xact [Abbott Vascular, USA], OptiMed Sinus Carotid [Optimed, Germany]. Stents ranged in diameter from 6 mm to 10 mm and in length from 20 to 40 mm. After stent placement, postdilatation with Savvy [Cordis, USA] and Trek [Abbott, USA] balloons with a maximum diameter of 5 mm was performed, if necessary (*n* = 153). A post-dilatation balloon diameter greater than 5 mm carries a higher risk of atherosclerotic plaque protrusion and no better haemodynamic effect. Atropine (0,5 mg IV bolus) application was used selectively, depending on the patient´s response to predilatation when bradycardia was below 40 beats per minute. Technically, the procedure was successful when a maximum of 30% residual stenosis was achieved. After removal of the protection device, a completion carotid arteriogram was performed. An orientation neurological examination was always performed before removal of the guiding sheath from the ACC. The femoral vascular access was treated in most cases with the AngioSeal closure device [Terumo, USA].

After the procedure, patients were transferred to a neurology or neurosurgical unit for observation, i.e. pulse and blood pressure measurement, puncture site inspection, and basic neurologic assessment, every 2 h for 24 h or until discharge. When a closing device had been used, full mobilization was allowed 6 hours later. Patients were discharged to home if no complications occurred within the first 24 h of observation.

### Outcomes

We investigated the demographic and clinical characteristics of patients undergoing CAS in Masaryk Hospital. The accompanying ailments that were analyzed were hypertension, high cholesterol, diabetes, coronary artery disease and reduced kidney function. Statin and anticoagulants use, and smoking were monitored.

Periprocedural complications were those, that occurred in the first 30 days after the procedure. Minor complications included: hypotension, bradycardia, short-term asystole, hypertension, dyspnea, decreased saturation, and vascular access complications (pseudoaneurysm). Transient ischemic attack was defined as a new transient neurological deficit lasting up to 24 h.

Serious complications included death, stroke and myocardial infarction. Stroke was defined as neurological deterioration of more than 24 h. We considered an ischemic stroke to be major if the worsened neurological condition persisted for 30 days or if it was the cause of death. A minor ischemic stroke was considered when it did not meet the criteria of a major stroke or TIA. We defined ipsilateral ischemic stroke and ICH as procedure-related strokes. Routine cardiac enzyme sampling was not performed, and myocardial infarction had to be associated with new clinical symptomatology. Assessment of clinical status was performed by the attending physician, a neurologist or neurosurgeon.

The 30-day procedure-related death and stroke rates were compared. Overall death, stroke, or myocardial infarction due to any cause in periprocedural period were also compared. At the same time, the rule was that if a patient had a stroke resulting in death, only death was counted in the overall total.

### Statistical analysis

Demographic variables were summarized with the use of descriptive statistics. Categorical variables were summarized as counts, percentage and difference were evaluated with χ^2^ test. Normality of data was tested by Kolmogorov–Smirnov normality test. Mann–Whitney U test, Kruskal–Wallis and difference of means test were used in further statistical analysis. STATISTICA software was used for statistical analysis.

## Results

### Patient and procedure characteristics

Over a nine-year period, we treated a total of 248 carotid arteries in 241 patients (74.3% men and 25.7% women) for carotid artery stenosis. In 234 patients only one side was treated endovascularly, and in seven patients both carotid arteries were treated. 114 stents were implanted in the right carotid bifurcation and four stents in the right common carotid artery (ACC). In the left carotid bifurcation were implanted 122 stents and seven in the left ACC. In one patient, ACC stenosis was resolved by angioplasty alone. Symptomatic stenosis was treated in 138 carotid arteries (55.6%), asymptomatic stenosis in 110 carotid arteries (44.4%). Primary atherosclerotic stenosis was present in 198 carotid arteries (79.8%) and carotid restenosis after previous CEA in 50 cases (20.2%).

All patients were set on in vitro efficient P2Y_12_ inhibitor as a part of DAPT. 167 patients (67.4%) were taking clopidogrel, 38 patients (15.3%) ticagrelor, 36 patients (14,5%) ticlopidine and 7 patients (2.8%) prasugrel. Platelet function tests revealed resistance to acetylsalicylic acid in 25.9% of patients (59/227). In twenty-one cases acetylsalicylic acid effectiveness was not evaluated.

The average age was 71 years, the youngest patient was 45 years old, the oldest 93 years old. The most common comorbidity in the whole cohort was arterial hypertension in 74% of patients, followed by dyslipidemia in 61% of patients, 35% of patients were treated for diabetes mellitus and half of patients are or were active smokers. Detailed patient demographics and characteristics of comorbidities are shown in Table 1. There was no statistically significant difference in the prevalence of any of the comorbidities studied between symptomatic and asymptomatic patients treated at MNUL. The characteristics of the patient cohorts from the CREST and ICSS randomized control trials are shown in Table 1.

**Table 1:** Demographics of patients undergoing CAS treatment in the Masaryk Hospital (MNUL), the CREST and the ICSS study^a^

	**Symptomatic patients MNUL (*n* = 138)**	**Asymptomatic patients MNUL (*n* = 110)**	**CREST (*n* = 1262)**	**ICSS (*n* = 853)**
**Mean age** ± SD	70.5 ± 9.0	71.7 ± 7.7	68.9 ± UD	70 ± UD
**Male sex** – no. (%)	102 (73.9%)	83 (75.5%)	63.9%	70%
**Hypertension** – no. (%)	98(72.0%)	81 (77.1%)	85.8%	69%
**Dyslipidaemia** – no. (%)	84 (61.76%)	64 (60.9%)	82.9%	61%
**Diabetes Mellitus** – no. (%)	45 (33.0%)	40 (38%)	30.6%	22%
**Atrial fibrillation** – no. (%)	15 (11.0%)	10 (9.2%)	UD	7%
**Coronary artery disease –** no. (%)	42 (30.8%)	30 (28.6%)	42.4%	UD
**Renal insufficiency** – no. (%)	12 (8.8%)	7 (6.6%)	UD	UD
**Smoking** – no. (%)	66 (48.5%)	53 (50.5%)	UD	72%
**Statin use** – no. (%)	96 (69.5%)	72 (65.5%)	UD	UD
**Anticoagulants use** – no. (%)	23 (16.6%)	7 (6.4%)	UD	UD

^a^*MNUL* Masaryk hospital, *CREST* Stenting versus Endarterectomy for Treatment of Carotid-Artery Stenosis, *ICSS* Carotid artery stenting compared with endarterectomy in patients with symptomatic carotid stenosis (International Carotid Stenting Study), *UD* indicate untraceable data

### Periprocedural complications

The most common non-serious complication in our cohort was hypotension in 6.5% and bradycardia in 2.4% of patients. Short-term transient asystole on postdilatation that did not require medical treatment occurred in 3 patients (1.2%). Hypertension also occurred in 3 patients (1.2%). In the asymptomatic group, the injection site was complicated by pseudoaneurysm formation in one patient. In only one patient the early periprocedural period was complicated by dyspnea. In one patient a decrease in saturation was noted. The total number of complications was greater in symptomatic patients (*n* = 19) than in asymptomatic patients (*n* = 11) (Table 2).

**Table 2:** Incidence of minor complications in MNUL

	**Whole cohort (*n* = 248)**	**Symptomatic cohort (*n* = 138)**	**Asymptomatic cohort (*n* = 110)**	**P**
**Hypotension**	16 (6.5%)	9 (6.5%)	7 (6.4%)	1
**Bradycardia**	6 (2.4%)	4 (2.9%)	2 (1.8%)	0,8932
**TIA**	3 (1.2%)	2 (1.8%)	1 (0.7%)	0,8430
**Hypertension**	3 (1.2%)	3 (2.2%)	0 (0%)	0,3314
**Asystole**	3 (1.2%)	2 (1.5%)	1 (0.9%)	1
**Pseudoaneurysm**	1 (0.4%)	0 (0%)	1 (0.9%)	1
**Dyspnoe**	1 (0.4%)	1 (0.7%)	0 (0%)	1
**Hyposaturation**	1 (0.4%)	1 (0.7%)	0 (0%)	1

[n (%)], *P* values were calculated by χ^2^ test

Overall, we observed six strokes in our cohort, including four major ischemic strokes, one minor ischemic stroke, and one intracranial hemorrhage (ICH). There were three ipsilateral disabling ischemic strokes (2.2%) in the symptomatic cohort. In the asymptomatic patient cohort, there was one minor ischemic stroke (0.9%) and one not procedure-related major ischemic stroke (0.9%).

A total of 5 patients died within 30 days of the procedure. One patient died of ICH on the 18th day after surgery and one patient died of ischemic stroke on the 30th day after surgery. The other three patients who died within 30 days were discharged in stable condition after CAS and the death was not directly related to the intervention. Two patients died of sepsis. One patient in the asymptomatic cohort had a middle cerebral artery occlusion in the contralateral carotid basin on day 8 after discharge, and despite a mechanical embolectomy, the stroke was fatal. In this case, we did not demonstrate an association with the procedure, and follow-up diffusion-weight magnetic resonance imaging after carotid stenting showed no ischemic changes. One patient had early stent occlusion on day 9 with subsequent severe ischemic stroke. Despite explicit instructions, the patient discontinued dual antiplatelet therapy after intervention because of noncompliance. We did not observe another stent occlusion. In the periprocedural period, we did not observe myocardial infarction (Table 3).

**Table 3:** Incidence of major complications

		**Total**	**Asymptomatic cohort [*n* = 110]**	**Symptomatic cohort [*n* = 138]**	**P**
**Major ischcemic stroke**	**ipsilateral**	3	0 (0%)	3 (2.2%)	1
	**non-ipsilateral**	1	1 (0.9%)	0 (0%)	1
**Minor ischemic stroke**	**ipsilateral**	1	1 (0.9%)	0 (0%)	1
	**non-ipsilateral**	0	0 (0%)	0 (0%)	NA
**Ischemic stroke**		5	2 (1.8%)	3 (2.2%)	1
**ICH**		1	0 (0%)	1 (0.7%)	1
**All stroke**		6	2 (1.8%)	4 (2.9%)	0,8932
**Procedure-related stroke**		5	1 (0.9%)	4 (2.9%)	0,5139
**Myocardial infarction**		0	0 (0%)	0 (0%)	NA
**All death**		5	1 (0.9%)	4 (2.9%)	0,5139
**Procedure – related death**		2	0 (0%)	2 (1.5%)	0,5801
**Procedure-related death or stroke**		5	1 (0.9%)	4 (2.9%)	0,4481
**Death, stroke, or IM**		8	2 (1.8%)	6 (4.3%)	0,4481
**Stent occlusion**		1	0 (0%)	1 (0.7%)	1

*ICH* intracerebral haemorrhage, *IM* myocardial infarction, *NA* denotes non applicable

*P* values were calculated by χ^2^ test

In the TIA cohort, two patients were taking DAPT with clopidogrel, one with ticagrelor. All patients had a Precise stent implanted. In the ischemic stroke cohort, four patients were taking DAPT with clopidogrel (2 × Precise stent, Wallstent and Adapt, respectively). One uncooperative patient discontinued ticagrelor with Wallstent implanted. The patient with intracerebral hemorrhage was taking DAPT with clopidogrel. Of the eight ischemic complications, six patients were taking clopidogrel, two were taking ticagrelor (1 × noncompliance). The Precise stent was implanted five times, Wallstent twice (1 × noncompliance) and Adapt once.

### Comparison of complications

In a cohort of asymptomatic patients (*n* = 110), we showed no statistically significant difference from RCTs in procedure-related death and stroke rate (0.9% in MNUL) or overall death, stroke, or IM rate (1.8% in MNUL). However, there is a trend towards a lower incidence of complications. Results comparing our cohort with individual studies are: CREST (0.9% vs. 2.5%, *P* = 0.1503; 1.8% vs. 3.6%, *P* = 0.1760), ACT-1 (0.9% vs. 3%, *P* = 0.2439; 1.8% vs. 3.3%, *p* = 0.1909), ACST-2 (0.9% vs. 3.8%, *P* = 0.1487; 1.8% vs. 3.9%, *P* = 0.1334) (Table 4).

**Table 4:** Comparison of selected complications in the asymptomatic patients

	**MNUL (*n* = 110)**	**SPACE-2 (*n* = 197)**	**ACST-2 (*n* = 1653)**	**ACT-I (*n* = 1032)**	**CREST (*n* = 594)**
**Procedure-related death or stroke**	1 (0.9%)	5 (2.5%)	62 (3.8%)	31 (3%)	15 (2.5%)
**Death, stroke, or IM**	2 (1.8%)	UD	65 (3.9%)	35 (3%)	21 (3.6%)

[n (%)], *IM* myocardial infarction, *MNUL* Masaryk hospital, *SPACE-2* Angioplasty in asymptomatic carotid artery stenosis vs. endarterectomy compared to best medical treatment, *ACST-2* Second asymptomatic carotid surgery trial: a randomized comparison of carotid artery stenting versus carotid endarterectomy, *ACT-1* Randomized Trial of Stent versus Surgery for Asymptomatic Carotid Stenosis, *CREST* Stenting versus Endarterectomy for Treatment of Carotid-Artery Stenosis. *UD* indicate untraceable data

In the cohort of symptomatic patients (*n* = 138), when comparing our cohort with the ICSS results, the procedure-related death or stroke was statistically significantly lower [2.9% vs 7.4%, *P* = 0.0243]. When comparing the overall death, stroke, or IM rate with those of the CREST and ICSS studies, there is again a trend towards a lower rate of complications, but not statistically significant [4.3% vs. 6.8%, *P* = 0.1393; and 4.3% vs. 7.4%, respectively] (Table 5).

**Table 5:** Comparison of selected complications in the symptomatic patients

	**MNUL (*n* = 138)**	**CREST (*n* = 668)**	**ICSS (*n* = 828)**
**Procedure-related death or stroke**	4 (2.9%)	40 (6%)	61 (7.4%)
**Death, stroke or IM**	6 (4.3%)	45 (6.8%)	61 (7.4%)

[n (%)], *IM* myocardial infarction, *MNUL* Masaryk hospital, *CREST* Stenting versus Endarterectomy for Treatment of Carotid-Artery Stenosis, *ICSS* Carotid artery stenting compared with endarterectomy in patients with symptomatic carotid stenosis (International Carotid Stenting Study)

Table 6 compares the entire MNUL, CREST and SAPPHIRE patient cohorts. There is a statistically significant difference in mortality and number of procedure-related strokes compared with the CREST trial [2.0% vs. 4.8%, *p* = 0.0243]. Overall death, stroke or IM rate were not statistically significant compared to CREST [3.2% vs. 5.8%, *P* = 0.513] or SAPPHIRE [3.2% vs. 4.4%, *P* = 0.2266].

**Table 6:** Comparison of selected complications in the entire cohorts of MNUL, CREST and SAPPHIRE studies

	**MNUL (*n* = 248)**	**CREST (*n* = 1144)**	**SAPPHIRE (*n* = 159)**
**Procedure-related death or stroke**	5 (2%)	55 (4.8%)	UD
**Death, stroke or IM**	8 (3.2%)	66 (5.8%)	7 (4.4%)

[n (%)], *IM* myocardial infarction, *MNUL* Masaryk Hospital, *CREST* Stenting versus Endarterectomy for Treatment of Carotid-Artery Stenosis, *SAPPHIRE* Protected Carotid-Artery Stenting versus Endarterectomy in High-Risk Patients. *UD* indicate untraceable data

## Discussion

Carotid stenting was introduced in the 1990s as a less invasive alternative for the treatment of carotid stenosis. There are only a few medical procedures that have been subjected to such rigorous and comprehensive scientific scrutiny. The results of large randomized controlled trials have demonstrated equivalence to carotid endarterectomy in periprocedural outcomes and long-term stroke prevention. Current American Heart Association (AHA) guidelines recommend center-based treatment of carotid stenosis when the risk of periprocedural stroke or death is ≤ 6% for symptomatic and ≤ 3% for asymptomatic stenoses [4–9, 25, 26].

Procedure-related death or stroke rate in our study was 2.0% and 2.9% for symptomatic and asymptomatic stenoses, respectively. The overall death, stroke or IM rate was 3.2% and 4.3% for symptomatic stenoses and asymptomatic stenoses, respectively. For both groups of patients, we fulfilled the established criteria even in our cohort of patients with higher surgical risk.

Historically, carotid stenting has been indicated in patients with high operative risk. Currently, a significant proportion of patients with carotid stenosis are candidates for both CEA and CAS. However, both treatment methods have their own risk factors that must be considered before a definitive treatment decision is made. A typical candidate for CAS is a patient with high operative risk from associated diseases such as heart failure, atherosclerotic involvement of coronary arteries, significant lung disease. Anatomical factors include poorly accessible lesions, post radiotherapy changes of the neck, contralateral carotid occlusion or recurrent laryngeal nerve palsy. Patients after previous carotid endarterectomy benefit more from carotid stenting. Similarly, there are limitations and risk factors associated with potential complications for CAS. Clinical factors include neurological and cognitive deterioration, recent ischemic stroke, bleeding disorders, renal insufficiency, and non-compliance or intolerance of antiplatelet therapy. Anatomic risk factors include significant tortuosity of the vessels, type III aortic arch, circumferential lesion calcification, and lesion-related thrombus [4, 25, 26]. Anatomic factors were the reason for technical failure in four of our patients. Assessment of these risk factors before or even during the procedure reduces the incidence of periprocedural complications.

The composition of periprocedural complications differs between CEA and CAS. While myocardial infarction and cranial nerve paresis are described to be more frequent in surgical treatment, thromboembolic complications in the form of stroke and TIA are predominant in endovascular treatment [4–11].

The incidence of procedure-related stroke in the RCTs we cited ranged from 2.8% to 7% for CAS, and from 1.4% to 3.1% for CEA. Myocardial infarction was reported in 0% to 2.4% of procedures in CAS, and in 0% to 6.1% in CEA. Cranial nerve paresis occurred in 45 of 857 patients during CEA and in no patients during CAS in the ICSS study. In the ACT-1 trial, this complication was observed in one of 1089 patients during CAS and in four of 364 during CEA [4–9]. The reduction in periprocedural minor strokes and TIAs can be described as another milestone in the development of carotid stenting [2, 3].

In the study by Huibers et al. evaluating procedure-related strokes occurring during the ICSS study, 74% of strokes occurred on the day of intervention in the CAS cohort (43/58). Of these, 34% occurred already during the procedure (20/58). In the CEA cohort, the rates were 40% (12/27) and 19% (5/27), respectively. Between day 1 and day 30, fifteen strokes occurred in both cohorts (CAS 26%, CEA 54%). 62% (36/58) and 41% (11/27) were non-invalidating in the CAS and CEA cohorts, respectively. Only half of the patients with stroke in the CAS cohort had arterial imaging and in 18% (5/27) were found stent occlusion [27]. The above shows that most of the complications of CAS occur in the first 24 h. Strokes are usually less severe compared to CEA and a significant proportion are due to stent occlusion. In our cohort, one ischemic stroke occurred after postdilatation, and two strokes occurred in the first seven hours after the procedure. One fatal ischemic stroke on the contralateral side occurred 3 days after the procedure. Nine days after the procedure, stent thrombosis and occlusion with intracranial embolization occurred in an uncooperative patient who stopped using antiplatelet therapy. We did not observe early stent thrombosis within 72 h after the procedure. TIA occurred in only three patients and manifested as transient hemiparesis during the procedure with subsequent ad integrum restoration.

The experience of the operator is a crucial factor for the success of CAS. In the ICSS study, two surgeons from two centers were excluded for causing 5 fatal or disabling strokes during 11 carotid interventions. It was equivalent to nearly 10% of strokes in the entire study [4]. CAS was performed by three experienced interventional radiologists with more than 10 years of practice.

Protective devices have been developed to prevent thromboembolic events. The proximal protection device is based on the principle of inflating an occlusion balloon and reversing the flow. Umbrella-like device acts as distal protection to capture downstream particles. The risk of embolization during CAS is highest during the stent release and postdilatation. There have not been proven significant difference in the reduction of clinically significant stroke between the two types of protective, probably because of the low incidence of these complications. Only two meta-analyses have associated the use of these devices with a reduced incidence of stroke and death [28, 29]. Failure of distal protection occurred in a single patient in our cohort with a stroke occurring during procedure. In this case, the atherosclerotic plaque or its fatty core embolized intracranially during postdilatation.

Several studies have shown encouraging results of CAS performed with double-layer stents, which aim to reduce the risk of plaque rupture [30]. The use of double-layer stents has reduced the incidence of periprocedural and late stroke in both symptomatic and asymptomatic patients [31]. We observed no complications in our subset of patients implanted with the double-layered CGuard stent (Inspire-MD, Tel Aviv, Israel). The incidence of this type of stent in our cohort was low (6.2%). Therefore, we do not consider the comparison with RCT´s to be affected.

Comparing results of entire cohort of this study to CREST and SAPPHIRE results, the risk of procedure-related death or stroke was statistically significantly lower in MNUL than in the CREST. In symptomatic patients the risk of procedure-related death or stroke was statistically significantly lower in MNUL than in the ICSS studies. In the group of symptomatic patients, platelet function testing reduced procedure-related death and stroke rate, but not significantly. In a group of asymptomatic patients with a lower complication rate, we do not have a large enough cohort to prove this claim. The results demonstrate that routine testing of antiplatelet therapy and subsequent adjustment of therapy is safe. Our data suggest that DAPT with clopidogrel is less effective in preventing ischemic complications than a modified DAPT regimen.

Optimal medical therapy including antiplatelet therapy, statins, antihypertensives in combination with a healthy lifestyle and smoking cessation is necessary part of carotid artery stenosis treatment [4]. In our opinion, medical therapy before the procedure is as important as the correct patient selection and safe performance of the procedure. All selected RCT were using DAPT during CAS and after CAS, however none were using tailored DAPT or proved efficiency of established therapy. Patients in CREST study received aspirin, at a dose of 325 mg twice daily, and clopidogrel at a dose of 75 mg twice daily at least 48 h before the procedure. If the interval was shorter, DAPT was intensified. After the procedure, patients received one or two 325-mg doses of aspirin daily for 30 days and either clopidogrel, 75 mg daily, or ticlopidine, 250 mg twice daily, for 4 weeks. The continuation of antiplatelet therapy for more than 4 weeks after the procedure was recommended for all patients who had undergone carotid-artery stenting [9]. In the ICSS study a combination of aspirin and clopidogrel to cover stenting procedures was only recommended, with no further details provided [5].

We required using DAPT with clopidogrel at least 5 days before the procedure. Implantation of thrombogenic material is associated with serious complications including stroke and even stent occlusion, if treatment is ineffective [18, 19, 27]. Consensus guidelines for DAPT in percutaneous coronary interventions recommend against clopidogrel in favor of the newer P2Y12 inhibitors prasugrel or ticagrelor [32, 33]. In our study, we limited the use of Prasugrel to asymptomatic patients due, as a history of stroke is listed as a contraindication. Ticagrelor is contraindicated when there is a history of intracranial hemorrhage [32, 33]. The use of both alternatives in carotid stenting is still not common in the Czech Republic, unlike in other countries. Recent publications confirm clopidogrel resistance as a potential predictive factor for thromboembolic complications in CAS and state ticagrelor as a suitable alternative [14–17, 34, 35].

At our institution, we routinely tested the efficacy of antiplatelet therapy prior to elective procedure to reduce the risk of stroke and stent thrombosis. One third of the patients in our study were resistant to clopidogrel and a therapy change was required. Of the 8 ischemic complications, 6 were with clopidogrel and 2 with ticagrelor (1 × noncompliance). Our data suggest that DAPT with clopidogrel is less effective in preventing ischemic complications than a tailored DAPT regimen, despite in vitro proven efficacy.

Medication has a major impact on the outcome of carotid stenting. This was confirmed by studies analysing tandem stenosis sets treated during the management of acute stroke without standard premedication with DAPT. In the multicenter study by Allard et al. stent occlusion occurred in 20% of cases within 36 h after the procedure. Stent occlusion was also associated with worse functional outcomes and a higher risk of death with stent occlusion. The use of P2Y_12_ antagonists in carotid artery stenting during treatment of acute stroke is also associated with longer stent patency and lower mortality [27]. Our unpublished data show the same occlusion rate for acutely implanted stents without DAPT. In the study by Lacman et al., three stents occluded in patients treated with ASA monotherapy alone. The closures were asymptomatic [26].

## Conclusion

In our cohort of patients unsuitable for surgical treatment of carotid stenosis, we tested the efficacy of antiplatelet therapy before carotid stenting. One-third of patients were resistant to clopidogrel and required a change to laboratory-effective therapy. The risk of procedure-related death or stroke was statistically significantly lower than in the CREST and ICSS studies. The results demonstrate that routine testing of antiplatelet therapy and subsequent adjustment of therapy is safe. In the group of symptomatic patients, platelet function testing reduced procedure-related death and stroke rate. Our data suggest that DAPT with clopidogrel is less effective in preventing ischemic complications than a modified DAPT regimen. Routine adjustment of effective antiplatelet therapy before each carotid artery stenting may be beneficial in reducing thromboembolic complications.

## Data Availability

The datasets used and/or analysed during the current study are available from the corresponding author on reasonable request.

## Abbreviations

CAS: Carotid artery stenting
CEA: Carotid endarterectomy
ACC: Common carotid artery
ACI: Internal carotid artery
DAPT: Dual antiplatelet therapy
MNUL: Masaryk Hospital in Ústí nad Labem
CREST: Stenting versus Endarterectomy for Treatment of Carotid-Artery Stenosis study
ICSS: Carotid artery stenting compared with endarterectomy in patients with symptomatic carotid stenosis (International Carotid Stenting Study)
ACST-2: Second asymptomatic carotid surgery trial: A Randomized Comparison of Carotid Artery Stenting versus Carotid Endarterectomy
SAPPHIRE: Protected Carotid-Artery Stenting versus Endarterectomy in High-Risk Patients
SPACE-2: Angioplasty in asymptomatic carotid artery stenosis vs. endarterectomy compared to best medical treatment
ACT-1: Randomized Trial of Stent versus Surgery for Asymptomatic Carotid Stenosis
RCT: Randomized control trials
IM: Myocardial infarct
TIA: Transitory ischemic attack
ICH: Intracerebral hemorrhage

## References

[1] Campbell BCV, et al. Ischaemic stroke Nature Reviews Disease Primers. 2019;5(1):70. 31601801 10.1038/s41572-019-0118-8

[2] Lamanna A, et al. Carotid artery stenting: current state of evidence and future directions. Acta Neurologica Scandinavia. 2019;139(4):318–33. 10.1111/ane.13062 30613950

[3] Al-Mubarak N, Roubin GS, Iyer SS, Vitek JJ. Carotid artery stenting: Current Practice and Techniques. Philadelphia: Lippincott Williams Wilkins; 2004. ISBN 0-7817-4385-0.

[4] White JCh, et al. Carotid Artery Stenting: a JACC State-of-the-Art Review. J Am Coll Cardiol. 2022;80(2):155–70. 10.1016/j.jacc.2022.05.007 35798450

[5] Bonati LH, Dobson J, Featherstone RL, et al. Long-term outcomes after stenting versus endarterectomy for treatment of symptomatic carotid stenosis: the International Carotid Stenting Study (ICSS) randomised trial. Lancet. 2015;385(9967):529–38. 10.1016/S0140-6736(14)61184-3 PMC4322188 25453443

[6] Halliday A, Bulbulia R,Bonati LH, et al. Second asymptomatic carotid surgery trial (ACST-2): a randomized comparison of carotid artery stenting versus carotid endarterectomy. Lancet. 2021;398:1065–73. 10.1016/S0140-6736(21)01910-3 PMC8473558 34469763

[7] Rosenfield K, Matsumura JS , Chaturvedi S, et al. Randomized trial of stent versus surgery for asymptomatic carotid stenosis. N Engl J Med. 2016;374(11):1011–20. 10.1056/NEJMoa1515706 26886419

[8] Reiff T, Eckstein HH, Mansmann U, et al. Angioplasty in asymptomatic carotid artery stenosis vs. endarterectomy compared to best medical treatment: one-year interim results of SPACE-2. Int J Stroke. 2020;15(6). 10.1177/1747493019833017 PMC7416333 30873912

[9] Brott TG, Howard G, Roubin GS, et al. Long-term results of stenting versus endarterectomy for carotid-artery stenosis. N Engl J Med. 2016;374(11):1021–23. 10.1056/NEJMoa1505215 PMC4874663 26890472

[10] White CJ, Ramee SR, Collins, et al. Carotid artery stenting: patient, lesion, and procedural characteristics that increase procedural complications. Catheter Cardiovasc Interv. 2013;82(5):715–26. 10.1002/ccd.24984 23630062

[11] Voeks JH, Howard G, Roubin GS, et al. Age and outcomes after carotid stenting and endarterectomy: the carotid revascularization endarterectomy versus stenting trial. Stroke. 2011;42(12):3884–90. 10.1161/STROKEAHA.111.624155 PMC3312471 21980205

[12] Howard G, Roubin GS, Jansen O, et al. Association between age and risk of stroke or death from carotid endarterectomy and carotid stenting: a meta-analysis of pooled patient data from four randomised trials. Lancet. 2016;387(10025):1305–11. 10.1016/S0140-6736(15)01309-4 26880122

[13] Li S, Li BM, Zhou DB, et al. Cause and treatment for intracranial hemorrhage during the perioperative period of carotid artery stenting. Zhonghua Wai Ke Za Zhi. 2010;48:582–4. 20646473

[14] Fifi JT, Brockington C, Narang J, et al. Clopidogrel resistance is associated with thromboembolic complications in patients undergoing neurovascular stenting. AJNR Am J Neuroradiol. 2013;34:716–20. 10.3174/ajnr.A3405. 23194833 10.3174/ajnr.A3405 PMC7964502

[15] Koh JS, Hwang G, Park JCH, et al. Tailored antiplatelet therapy in stent-assisted coiling for unruptured aneurysms: a nationwide registry study. Journal of NeuroInterventional Surgery [Published online first: 03 January 2023]. 10.1136/jnis-2022-019571. 10.1136/jnis-2022-019571 36596671

[16] Li W, Zhu W, Wang A, Zhang G, et al. Effect of Adjusted Antiplatelet Therapy on Preventing Ischemic Events After Stenting for Intracranial Aneurysms. Stroke. 2021;52(12). 10.1161/STROKEAHA.120.032989. 10.1161/STROKEAHA.120.032989 34538087

[17] Simonte G, Guglielmini G, Falcinelli E, et al. High-on-treatment platelet reactivity predicts adverse outcome after carotid artery stenting: a prospective study. Thromb Res. 2023:22:117–23. 10.1016/j.thromres.2022.12.015 36640567

[18] Collette LS, Bokers RPH, Dierckx RAJO, et al. Clinical importance of testing for clopidogrel resistance in patients undergoing carotid artery stenting-a systematic review. Ann Transl Med. 2021;9(14):1211. 34430652 10.21037/atm-20-7153 PMC8350701

[19] Muram S, Panchendrabose K, Eagles ME, et al. Natural history of antiplatelet nonresponders undergoing carotid artery stenting. J Neurosurg. 2023;139(3):661–9. 10.3171/2022.12.JNS222316 36708530

[20] Vigláš P, Cihlář F, Bradáčová P, et al. Testing the efficacy of antiplatelet therapy - a single centre experience. Ces Radiol 2023;77(1):35–10.

[21] Kim KS, Fraser JF, Grupke S, Cook AM. Management of antiplatelet therapy in patients undergoing neuroendovascular procedures. J Neurosurg JNS. 2018;129(4):890–905. 10.3171/2017.5.JNS162307 29192856

[22] Krishnan K, Nguyen TN, Appleton JP, et al. Antiplatelet Resistance: A Review of Concepts, Mechanisms, and Implications for Management in Acute Ischemic Stroke and Transient Ischemic Attack. Stroke Vasc Interv Neurol. 2023;3:e000576. 10.1161/SVIN.122.000576 PMC12778636 41584975

[23] Dorsam RT, Kunapuli SP. Central role of the P2Y12 receptor in platelet activation. J Clin Investig. 2004;113(3):340–5. 10.1172/JCI20986. 14755328 10.1172/JCI20986 PMC324551

[24] Jay SY, Wholey MH, Kuntz RE, et al. Protected Carotid-Artery Stenting versus Endarterectomy. N Engl J Med. 2004;351:15. 10.1056/NEJMoa040127 15470212

[25] Lojík M, Krajíčková D, Kubíková M, et al. Endovascular treatment of carotid artery stenosis using cerebral protection: five-year experience. Discussion. Chir. 2007;86(10):513–20. 18064788

[26] Lacman J, Charvát F, Mašková J, Belšan T, Mohapl M. Treatment of atherosclerotic stenosis of the carotid bifurcation: seven-year experience from one institution. Ces Radiol. 2009;63(2):113–21.

[27] Huibers A, Calvet D, Kennedy F, et al. Mechanism of Procedural Stroke Following Carotid Endarterectomy or Carotid Artery Stenting Within the International Carotid Stenting Study (ICSS) Randomised Trial. Eur J Vasc Endovasc Surg. 2015;50:281e288. 26160210 10.1016/j.ejvs.2015.05.017 PMC4580136

[28] Touze E, Trinquart L, Chatellier L, Mas JL. Systematic review of the perioperative risks of stroke or death after carotid angioplasty and stenting. Stroke. 2009;40(12):683–93. 10.1161/STROKEAHA.109.562041 19892997

[29] Garg N, Karagiorgos N, Pisimisis GT, et al. Cerebral protection devices reduce periprocedural strokes during carotid angioplasty and stenting: a systematic review of the current literature. J Endovasc Ther. 2009;16(4):412–27. 10.1583/09-2713.1 19702342

[30] Stabile E, De Donato G, Musialek P, et al. Use of Dual-Layered Stents for Carotid Artery Angioplasty: 1-Year Results of a Patient-Based Meta-Analysis. JACC: Cardiovasc Intervent. 2020;13(14):1709–15. 10.1016/j.jcin.2020.03.048 32703595

[31] Sýkora J, Zeleňák K, Vorčák M, at al. Benefits and pitfalls of carotid stents with double-layer design - a systematic review. Cesk Slov Neurol N. 2023;86(3):171–6.

[32] Galli M, Benenati S, Capodanno D, et al. Guided versus standard antiplatelet therapy in patients undergoing percutaneous coronary intervention: a systematic review and meta-analysis. Lancet. 2021;397:1470–83. 33865495 10.1016/S0140-6736(21)00533-X

[33] Valgimigli M, Bueno H, Byrne RA, et al. ESC Scientific Document Group; ESC Committee for Practice Guidelines (CPG); ESC National Cardiac Societies. 2017 ESC focused update on dual antiplatelet therapy in coronary artery disease developed in collaboration with EACTS: the Task Force for dual antiplatelet therapy in coronary artery disease of the European Society of Cardiology (ESC) and of the European Association for Cardio-Thoracic Surgery (EACTS). Eur Heart J. 2018;39:13–260.

[34] Lotan D, Itsekzon-Hayosh Z, Itelman E, et al. Safety and efficacy of ticagrelor in carotid artery angioplasty in patients with clopidogrel resistance: real life experience. J Am Coll Cardiol. 2020;75(11 Suppl 1):1302.

[35] Allard J, Delvoye F, Pop R, at al. 24-Hour Carotid Stent Patency and Outcomes After Endovascular Therapy: A Multicenter Study. Stroke. 2023;54:124–31. 10.1161/STROKEAHA.122.039797 36542074

